# Development of one-step quantitative reverse transcription PCR for the rapid detection of flaviviruses

**DOI:** 10.1186/1743-422X-10-58

**Published:** 2013-02-14

**Authors:** Pranav Patel, Olfert Landt, Marco Kaiser, Oumar Faye, Tanja Koppe, Ulrich Lass, Amadou A Sall, Matthias Niedrig

**Affiliations:** 1 Robert Koch Institute, Center for Biological Security 1 (ZBS1), Nordufer 20, Berlin, 13353, Germany; 2 TIB MOLBIOL Syntheselabor, Berlin, Germany; 3 GenExpress, Berlin, Germany; 4 Institut Pasteur de Dakar, Dakar, Senegal

**Keywords:** Pan-Flavi assay, Locked-nucleic acid, Flaviviruses, qRT-PCR

## Abstract

**Background:**

The genus *Flavivirus* includes several pathogenic agents that cause severe illness in humans. Re-emergence of West Nile virus in Europe and continuous spread of certain flaviviruses such as dengue, yellow fever and Japanese encephalitis viruses represent a global danger to public health. Therefore, a rapid and accurate molecular method is required for diagnostics and epidemiological surveillance of flaviviruses.

**Methods:**

A Pan-Flavi quantitative RT-PCR assay using a Locked-Nucleic Acid probe targeting the flavivirus NS5 gene was developed and optimized to detect a wide range of flaviviruses simultaneously. The specificity and sensitivity of the Pan-Flavi assay were tested using RNA of different flaviviruses and non-flaviviruses. Furthermore, the assay was compared directly to flavivirus species-specific assays for the ability to detect flaviviruses sensitively.

**Results:**

Two degenerate primers and one Locked-Nucleic Acids probe were designed to amplify most of the flaviviruses. To increase the specificity and fluorescence signal of the Pan-Flavi assay for detection of yellow fever virus and dengue virus 4, additional primers and probes were included. Viral RNA of thirty different flaviviruses was detected, verifying the broad range specificity. The testing of this assay was successful, using standard plasmid and RNA dilutions of yellow fever virus vaccine strain, dengue virus 1 and tick-borne encephalitis virus, with a sensitivity limit of 10–100 genome copies/reaction. Also comparatively good results were achieved for detecting different flaviviruses by the Pan-Flavi assay when compared to the flavivirus species-specific assays.

**Conclusion:**

The assay is rapid, broad-range flavivirus-specific and highly sensitive making it a valuable tool for rapid detection of flaviviruses in livestock samples, epidemiological studies or as useful complement to single flavivirus-specific assays for clinical diagnosis.

## Background

The genus *Flavivirus* of the family *Flaviviridae* consists of more than 70 virus species including many arthropod-borne viruses. It contains highly pathogenic agents such as the name-giving member yellow fever virus (YFV), West Nile virus (WNV), Japanese encephalitis virus (JEV), tick-borne encephalitis virus (TBEV) and dengue virus (DENV), which can cause encephalitis or hemorrhagic fever and pose some of the most challenging emerging diseases in humans [1].

Flaviviruses comprise three epidemiologically distinct groups; the mosquito-borne group, the tick-borne group and the unknown vector viruses [2]. They are enveloped positive-stranded RNA viruses with a genome of approximately 11 kb. The viral genome encodes a large single polyprotein from which three structural proteins, Capsid (C), Envelope (E) and Membrane (M) and seven non-structural proteins, NS1, NS2A, NS2B, NS3, NS4A, NS4B and NS5, are produced [3].

Flaviviruses pose a threat to public health worldwide, especially in developing countries. In recent years, there has been a dramatic increase in cases of DENV infections globally. The disease is endemic in more than 100 countries in Africa, the Americas, the Eastern Mediterranean, South-east Asia, and the Western Pacific. About 50–100 million cases of DENV infection are reported worldwide every year, resulting in 500,000 cases of hemorrhagic fever requiring hospitalization and 25,000 fatalities [4]. Yellow Fever is endemic in 45 countries throughout Africa and Latin America, with approximately 200,000 cases of human infections worldwide, and 30,000 mortalities [5]. WNV is one of the most widespread flaviviruses. WNV lineage 1 is endemic in the Americas, India, and Australia. Both lineages 1 and 2 are found in Africa and Europe [6-8]. The recent WNV outbreak reported in August 2010 in Greece was the first large outbreak in humans in Europe since the Romanian outbreak in 1996–1997 [9]. JEV is the leading cause of viral encephalitis in Asia with 30,000–50,000 clinical cases reported annually and 10,000 deaths [10]. TBEV, virus-causing infection of the central nervous system, has spread extensively in some European regions. According to ECDC [11], human cases of TEBV infections have increased in the past 30 years dramatically posing a danger to public health in European countries.

A number of flaviviruses have repeatedly demonstrated their ability to expand their geographical range. Some current examples are the introduction of Usutu virus and Bagaza virus into central Europe [12,13], or Zika virus into Yap island and Cambodia [14]. In recent years, also a high number of mosquito-only flaviviruses including *Culex* and *Aedes* flaviviruses were discovered throughout the world [15-18]. Apart from the human pathogenic and mosquito-only flaviviruses, several novel flaviviruses were latterly discovered: Nounane in Cote d’Ivoire [19], T’Ho virus in Mexico [20], Lammi virus in Finnland [21], and Duck tembusu virus in China [22]. In contrast to mosquito-only flaviviruses, these novel flaviviruses were shown to be phylogenetically related to human pathogenic flaviviruses.

The clinical diagnosis of flavivirus infections is not unambiguous due to unspecific symptoms varying from mild, febrile illness to viral hemorrhagic fever. Many of these viruses have a common transmission vector and can co-circulate in the same area. All these factors make a precise identification of the pathogen difficult.

Today most diagnostic laboratories use serological assays for flavivirus testing. These tests are commonly based on the enzyme-linked immunosorbent assay (ELISA) and detect virus-specific IgM and IgG antibodies. Antibodies are undetectable prior to 5–7 days after onset of infection which hampers the usefulness of the serological methods. Molecular techniques, in contrast, can be used already in the acute phase and are known to be rapid, highly specific and sensitive.

Since the early 1990s, several group-specific and generic molecular assays for detection of flaviviruses have been developed as reviewed previously [23-25]. A number of attempts to detect several flaviviruses in a single reverse transcription-polymerase chain reaction (RT-PCR) assay have been made [26]. These assays vary in the selected target region, assay format and detection method. The highly conserved NS3 and NS5 genes have been used predominantly for flavivirus testing, mainly as nested, hemi-nested RT-PCR or SYBR green real-time PCR, without using a probe for sequence verification.

The aim of this study was to develop a rapid, sensitive and reliable TaqMan probe-based quantitative RT-PCR (qRT-PCR) assay for simultaneous detection of several flaviviruses, using the conserved NS5 gene region. The introduction of Locked-Nucleic Acid (LNA) bases in the probe increases robustness, specificity and sensitivity of the assay and has been shown to allow quantification of the viral load.

## Results

### Design and optimization of Pan-Flavi assay

Primers from publication [26-28] were aligned against genomic sequences of all flaviviruses deposited in the NCBI database to verify their flavivirus-generic amplification capacity. Degenerate primers were selected based on these published sequences, but modified in order to increase the spectrum of flaviviruses included. As alternative approach individual specific flavivirus species-specific primers were combined to avoid unspecificity due to base combinations contained in the degenerate primers not matching exisiting viral sequences. In particular we used 17 forward and 15 reverse primers and another four reverse primers targeting different locations. Testing of the primer mix with viral RNA revealed that the performance of the primer mixes was not improved compared to the results obtained with the degenerate primers. Also for the probes we tested 22 individual flavivirus-specific sequences covering all known sequence variants at the binding site and established mixtures yielding comparable threshold cycle (Ct) values for all virus species tested, but the signal level was significant lower than for degenerate LNA-probes (data not shown). In conclusion, neither the set of individual primers nor the set of probes improved the detection limit nor the range of viruses detected and we continued the evaluation with the degenerate set of primers combined with one of the three LNA-probes.

In total, six combinations assembled from two degenerate primer sets (Flavi all S & Flavi all AS1 and Flavi all S & Flavi all AS2) and three probes (Flavi all probe 1, Flavi all probe 2 and Flavi all probe 3) were tested using RNA from flaviviruses and other viruses to check the performance of these Pan-Flavi assays. Combinations with Flavi all probe 3 showed earlier Ct values (Table 1). Best results among these six combinations were obtained with Flavi all S, Flavi all AS2 primers and Flavi all probe 3, but detection of DENV type 4 was insufficient. Analysis by gel electrophoresis revealed missing amplification, except a faint band for primer combination Flavi all S and AS1, correlating to a low signal and very late Ct value (Table 1).

**Table 1:** Comparison of designed two degenerate primer sets and three probes containing wobble and LNA bases in six combinations for detection of the different flavivirus RNA

***Sample No***	***Virus***	***Ct value obtained through testing the combination of Flavi all degenerate primers and probe***					
		**S/AS1/probe1**	**S/AS1/probe2**	**S/AS1/probe3**	**S/AS2/probe1**	**S/AS2/probe2**	**S/AS2/probe3**
**1**	DENV-1	31.50	17.57	15.66	27.95	17.84	15.61
**2**	DENV-2	22.69	22.21	19.76	22.27	22.19	19.92
**3**	DENV-3	No Ct	21.25	19.14	No Ct	20.63	18.57
**4**	DENV-4	No Ct	35.50	35.48	No Ct	No Ct	No Ct
**5**	JEV	20.40	21.63	20.93	18.99	19.98	19.27
**6**	RSSEV	27.69	29.38	27.31	24.67	26.13	23.81
**7**	TBEV K23	17.68	21.81	16.99	16.73	18.63	16.32
**8**	TBEV Louping ill	26.00	28.75	23.78	22.13	24.8	21.22
**9**	Usutu virus	25.63	33.96	25.91	22.96	30.3	23.48
**10**	WNV Uganda	30.22	30.27	30.68	28.46	28.45	28.26
**11**	WNV Israel	21.76	21.83	20.78	20.06	21.68	19.8
**12**	SLEV	25.85	26.95	25.57	24.41	24.90	23.49
**13**	YFV 17D	19.82	22.48	18.41	20.03	22.12	18.92
**14**	YFV Asibi	23.71	26.98	22.82	24.17	26.83	23.53
**15**	YFV Brasil	21.35	23.73	20.48	21.75	23.82	21.43
**16**	YFV Ivory Coast	21.35	23.98	20.36	21.47	23.94	21.44
**18**	Chikungunya virus	No Ct	No Ct	No Ct	No Ct	No Ct	No Ct
**19**	RVFV	No Ct	No Ct	No Ct	No Ct	No Ct	No Ct
**20**	Sindbis virus	No Ct	No Ct	No Ct	No Ct	No Ct	No Ct
**21**	H1N1 Influenza A	No Ct	No Ct	No Ct	No Ct	No Ct	No Ct
**22**	H5N1 Influenza A	No Ct	No Ct	No Ct	No Ct	No Ct	No Ct
**17**	Negative control	No Ct	No Ct	No Ct	No Ct	No Ct	No Ct

*DENV*: Dengue virus, *JEV*: Japanese encephalitis virus, *RSSEV*: Russian spring-summer encephalitis virus, *WNV*: West Nile virus, *TBEV*: Tick-borne encephalitis virus, *SLEV*: Saint Louis encephalitis virus, *RVFV*: Rift valley fever virus, *YFV*: Yellow fever virus.

S: Flavi all S, AS1: Falvi all AS1, AS2: Flavi all AS2, probe1: Flavi all probe 1, probe 2: Flavi all probe 2, probe 3: Flavi all probe 3.

To overcome this deficit the combination Flavi all S/AS2/probe 3 was supplemented with an additional DEN4 F primer and all tests were repeated. DENV type 4 was now detectable with Ct values similar to the specific assay. The additional DEN4 F primer had no influence on the detection of all other flaviviruses tested (Table 2). Evaluation of the Pan-Flavi assay was performed using primers Flavi all S/DEN4 F and Flavi all AS2. Changing primers to Flavi all S/Flavi all S2/ Flavi all AS4 allowed to detect also Nounane virus (Table 3), without changing specificity and sensitivity of Pan-Flavi assay for the other viruses (data not shown).

**Table 2:** Representative results of the Pan-Flavi assay to detect different flaviviruses, and its comparison with flavivirus species-specific assays

**Virus**	**Strain/ Acc. No.**	Pan-flavi assay*^,#^			**Virus-specific assay**^**#**^			
		**Mean of Ct value**	**SD of Ct value**	**Mean of GC/ml**	**Mean of Ct value**	**SD of Ct value**	**Mean of GC/ml**	**Reference**
***Flavivirus***								
DENV-1	ATCC VR-344	15.64	0.02	2.0 × 10^9^	15.93	0.03	4.6 × 10^9^	[29]
DENV-2	ATCC VR-345	20.76	0.20	6.6 × 10^7^	18.77	0.11	6.7 × 10^8^	
DENV-3	ATCC VR-1256	19.36	0.01	1.7 × 10^8^	20.30	0.01	2.4 × 10^8^	
DENV-4	ATCC VR-1257	16.33	0.19	1.3 × 10^9^	16.18	0.29	3.9 × 10^9^	
YFV 17D	X03700	19.17	0.17	1.9 × 10^8^	20.51	0.17	5.6 × 10^8^	[30]
YFV ASIBI	RKI reference strain	23.73	0.05	9.1 × 10^6^	23.72	0.28	4.7 × 10^7^	
YFV Brazil	RKI reference strain	21.18	0.07	5.0 × 10^7^	22.50	0.10	1.2 × 10^8^	
YFV Ivory Cost	RKI reference strain	21.44	0.21	4.2 × 10^7^	21.29	0.05	3.1 × 10^8^	
WNV Uganda	AY532665, lineage 1	28.74	0.06	3.2 × 10^5^	26.83	0.27	3.4 × 10^6^	[31]
WNV Israel	lineage 2	19.81	0.15	1.2 × 10^8^	19.86	0.01	4.0 × 10^8^	
TBEV K23	AF091010	16.33	0.03	1.3 × 10^9^	16.78	0.14	2.1 × 10^8^	[32]
RSSEV	RKI reference strain	24.20	0.32	6.7 × 10^6^	19.35	0.01	4.1 × 10^6^	
TBEV Louping ill	RKI reference strain	20.59	0.22	7.4 × 10^7^	20.43	0.06	2.0 × 10^7^	
JEV	ATCC SA14-14-2	19.36	0.01	2.4 × 10^8^	16.73	0.01	7.7 × 10^8^	In house assay

*GC*: genome copies, *Ct*: threshold cycle, *SD*: standard deviation.

*DENV*: Dengue virus, *JEV*: Japanese encephalitis virus, *RSSEV*: Russian spring-summer encephalitis virus, *WNV*: West Nile virus, *TBEV*: Tick-borne encephalitis virus, *YFV*: Yellow fever virus.

^*^Pan-Flavi assay was performed using Flavi-all S, Flavi all AS2, DEN4 F primer and Flavi all probe3 mix.

^#^The same amount of RNA (5 μl) was applied to both pan-Flavi assay and virus species-specific one-step qRT-PCR assays.

**Table 3:** Performance of the Pan-Flavi assay to detect different flaviviruses. Five μl of viral RNA extracted from virus cell culture supernatant were applied to both pan-Flavi assay and RT-PCR

**Virus**	**Virus abbreviation**	**Strain/Reference**	**Pan-flavi assay**	RT-PCR *
			**Ct value**	**Confirmation by gel**
***Flaviviridae***				
St. Louis Encephalitis virus	SLEV	ATCC VR-1265	22.19	N.d.
Usutu virus	USUV	AY453411	22.48	N.d.
Kunjin virus	KUNV	PI.France	24.97	N.d.
Kedougou virus	KEDV	ArD 14701	29.62	Positive
Koutango virus	KOUV	AnD 5443	30.29	Positive
Spondweni virus	SPOV	SA Ar 94	26.02	Negative
Uganda S virus	UGSV	ArD 109325	29.48	Positive
Wesselsbron	WESSV	ArB 4177	24.73	Positive
Zika virus	ZIKV	PI, Senegal	22.45	Positive
Sepik virus	SEPV	MK 7148	16.77	Positive
Dakar bat virus	DBV	AnD 249	32.57	Positive
Yaounade virus	YAOV	ArY 276/68	17.52	Positive
Bouboui virus	BOUV	ArB 490	31.88	Negative
Saboya virus	SABV	AnD 4600	27.50	Negative
Nounane virus^#^	NOUV	B3 isolate	No ct	N.d.
***Bunya viridae***				
Rift valley fever virus	RVFV	RKI	No ct	N.d.
***Togaviridae***				
Chikungunya virus		LR 2006	No ct	N.d.
Chikungunya virus		ST05,African isolate	No ct	N.d.
Sindbis virus		RKI	No ct	N.d.
***Influneza virus***				
H1N1 Hamburg		A/Hamburg/04/2009 H1N1	No ct	N.d.
H1N1 California		A/California/04/2009 H1N1	No ct	N.d.
H5N1		A/dk/Germany R603/06 H5N1	No ct	N.d.

*N.d*.: not done, *Ct*: threshold cycle, * RNA of African flaviviruses were detected using reference method described previously [33].^#^ Nounane virus was detected using final optimized primer sets Flavi all S/Flavi all S2/Flavi all AS4 and Flavi all probe 3 mix.

### Specificity evaluation of the Pan-Flavi assay

The specificity of the final version of the Pan-Flavi assay was tested using a panel of mosquito- and tick-borne flaviviruses, one insect-only novel flavivirus and one flavivirus with unknown vector, one virus of the *Bunyaviridae* family, three of the *Togaviridae* family and three of the *Orthomyxoviridae* family*.* Viral RNA of thirty different flaviviruses was detected, verifying the broad range specificity. However, one insect-only novel flavivirus was not detected. Neither non-flaviviruses nor negative controls resulted in signals, demonstrating the absence of cross-reactivity of the Pan-Flavi assay with non-flaviviruses (Tables 2 and 3).

### Sensitivity evaluation of the Pan-Flavi assay

**Figure 1:**
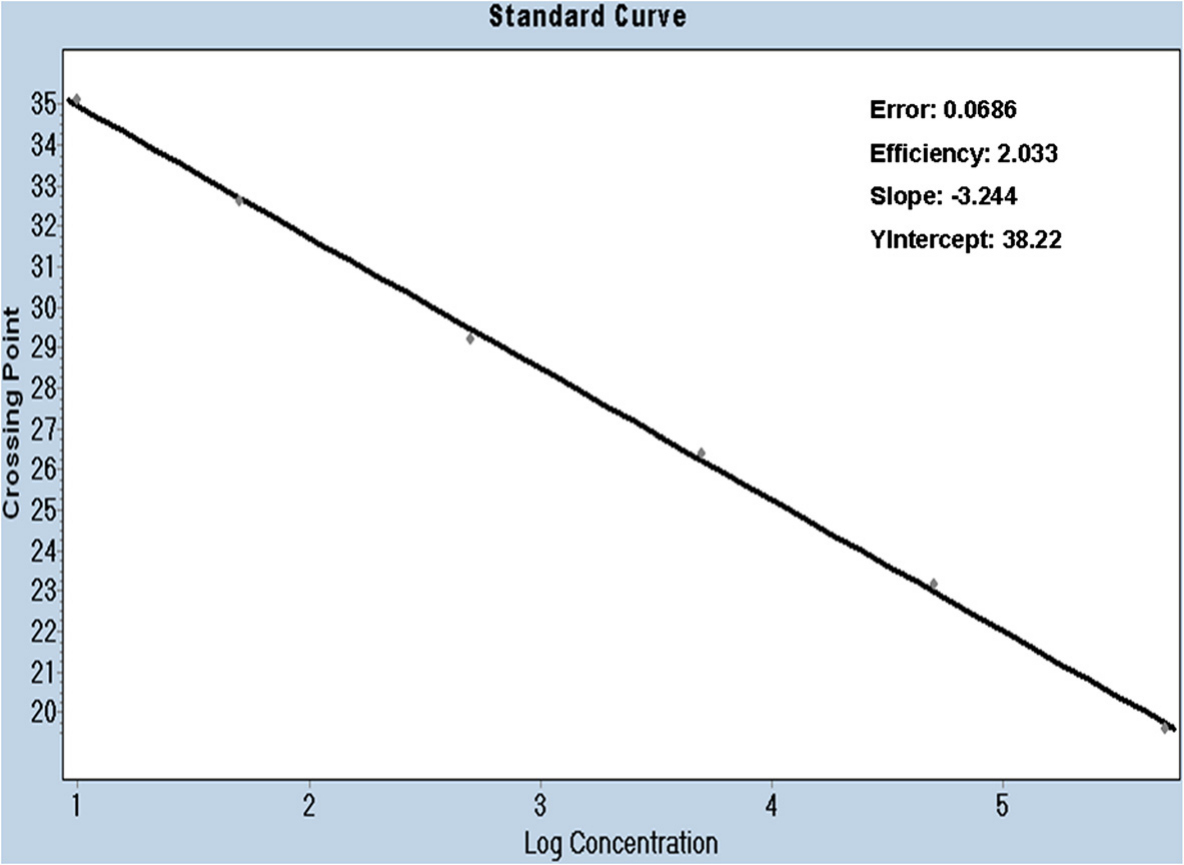
**Standard curve constructed using ten-fold serial dilution of standard plasmid containing NS5 gene of yellow fever virus.** The detection thresholds (Ct values) are plotted against the log of known standard plasmid (pYFV) copy numbers.

To determine the linearity of the assay, 10-fold serial dilutions of the standard plasmid were tested. The Pan-Flavi assay was shown to be linear over a range of 5 log_10_ copies. Limit of detection is at least 10 copies/reaction of standard plasmid (Figure 1).

The Pan-Flavi assay was performed as a one-step qRT-PCR. The sensitivity of the Pan-Flavi assay to detect viral RNA was compared to three virus species-specific one-step qRT-PCR assays for DENV, TBEV and YFV, all assays could be thus compared in one run. The RNA stocks of DENV-1, YFV strain 17D and TBEV strain K23 were quantified by virus species-specific qRT-PCR as described previously [29,30,32]. Then, 10-fold serial dilution of the RNA was prepared from quantified stocks to cover a range of 10^1^ to 10^7^ genome copies/reaction. The detection limit of Pan-Flavi assay was determined using these pre-quantified RNA dilutions. Detection limit of the Pan-Flavi assay was 100 genome copies for YFV 17D, 10 genome copies for DENV-1 and 10 genome copies for TBEV K23 (Table 4).

**Table 4:** Test of sensitivity of Pan-Flavi assay with pre-quantified viral RNA dilutions

**Quantity (Genome copies/reaction)**	**Ct values for DENV-1**	**Ct values for TBEV strain K23**	**Ct values for YFV strain 17D**
1 × 10^7^	16.8	16.6	-
1 × 10^6^	20.5	19.5	20.8
1 × 10^5^	23.4	23.0	24.2
1 × 10^4^	27.4	26.2	27.6
1 × 10^3^	30.2	29.4	30.1
1 × 10^2^	37.1	31.7	36.4
1 × 10^1^	39.7	33.0	No Ct

*GC*: genome copies, *Ct*: threshold cycle.

*YFV*: Yellow fever virus, *TBE*: Tick-borne encephalitis virus, *DENV*: Dengue virus.

### Comparison of the Pan-Flavi assay with virus species-specific qRT-PCR

The same amounts of RNA (5μl) from DENV, WNV, YFV, TBEV and JEV were detected quantitatively by using virus species-specific qRT-PCR assays, and the results were compared with those obtained by the Pan-Flavi assay for these viruses (Table 2). All four serotypes of DENVs were detected by the Pan-Flavi assay, with almost identical Ct values and GC/ml; although for DENV the Pan-Flavi assay showed delayed Ct values corresponding to one log dilution. For all YFV isolates tested the results were similar to those with the YFV-specific assay. Also WNV lineage 2 gave the same results as the WNV-specific assay, while the Ct values for lineage 1 virus were delayed. All three subtypes of TBEV were detected by both the Pan-Flavi assay and a TBEV-specific assay. The Ct values and genome copies/ml determined by both methods were identical except for RSSEV. For the detection of JEV, the Pan-Flavi assay had half a log lower sensitivity compared to the JEV-specific assay. WNV- and JEV-specific assays were performed in a two-step format which could explain their higher sensitivity compared to the Pan-Flavi assay.

In summary, the performance of the Pan-Flavi assay was comparable to virus species-specific assays except for minor differences for some strains of flaviviruses.

## Discussion

The ongoing emergence of flaviviruses poses major public health concerns worldwide. Techniques used for molecular testing and surveillance of flaviviruses should be able to detect and identify a wide range of flavivirus species rapidly, with a high level of specificity and sensitivity. For this purpose, an LNA probe-based qRT-PCR (Pan-Flavi assay) for detection of RNA of various flaviviruses was developed. We tested several important human-pathogenic flaviviruses to demonstrate the ability to detect relevant members of the flavivirus genus. The Pan-Flavi assay was also tested with other viruses, in particular phleboviruses, alphaviruses and influenza viruses, to determine absence of cross-reactivity.

In recent years, generic approaches targeting highly conserved NS3 and NS5 genes have been described for the detection of flaviviruses as summarized previously [24,26]. These methods were predominantly in the format of nested RT-PCR or SYBR green qRT-PCR which required post-amplification methods such as sequencing or melting temperature analysis to verify the result. In general, these methods have shown variable or lower sensitivity than species-specific approaches when evaluated during EQA exercises for the molecular diagnosis of different flavivirus [29,34]. Only in some cases assays were comparable to those from the species-specific methods [27].

In the past 10 years, the primer sets targeting a conserved region of NS5 have been developed and optimized for a broad detection of flaviviruses. This approach was not only useful to detect known flaviviruses, but has also led to discovery of novel flaviviruses [18,35,36].

**Figure 2:**
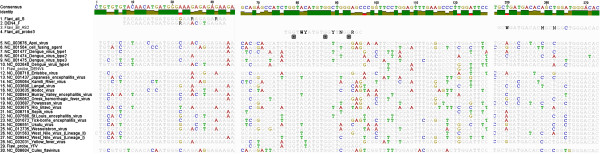
**Sequence alignment of primers and probe with 26 flaviviruses conserved region of NS5 gene.** + Symbol under oligonucleotides indicates position of locked nucleotides.

**Figure 3:**
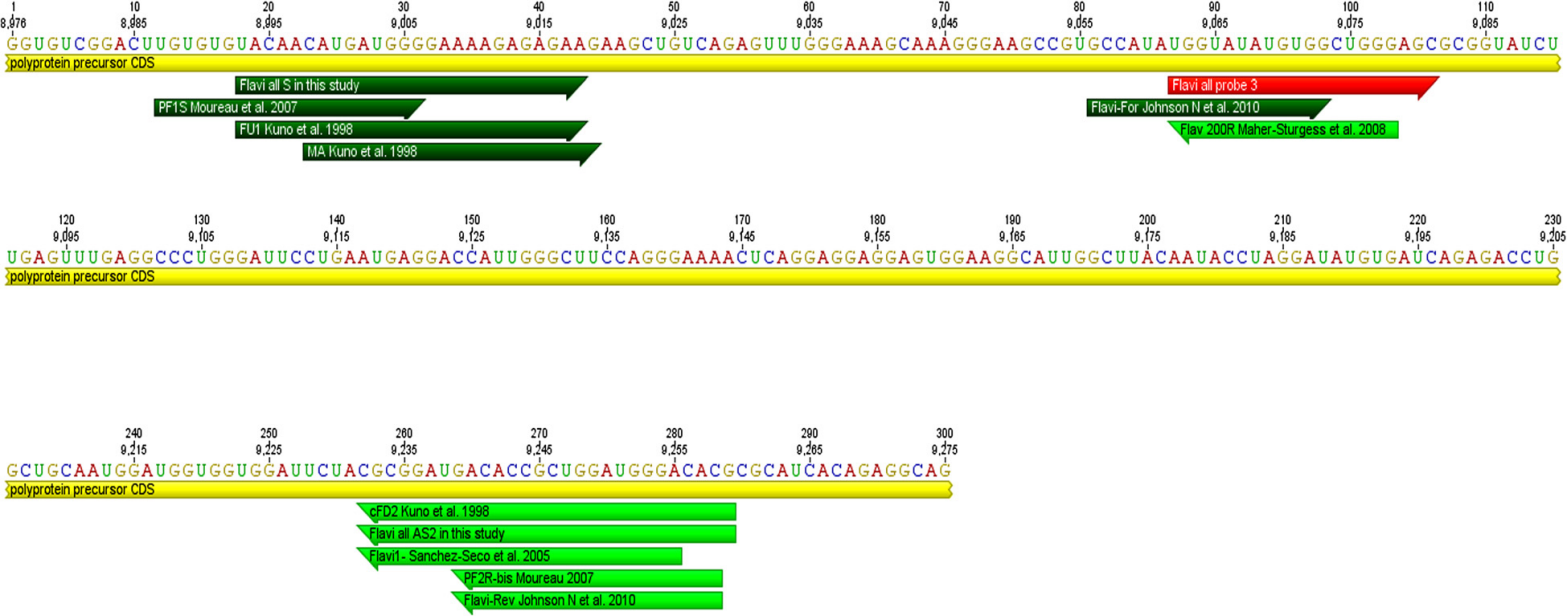
**The published and Flavi-all primer sites in the Yellow fever 17D genome (NC 002031) with locations of genes.** In dark green: forward primer, in light green: reverse primer, in red: site of probe used in this study. The figure is prepared using Geneious pro version (5.4.3) created by Biomatters.

For the novel Pan-Flavi assay, the degenerate primer sequences share the conserved NS5 region (Figure 2) targeted by the previous published primers (Figure 3). These primers exhibited good efficiency in detecting a broad range of common and exotic flaviviruses (Tables 2 and 3) but failed to detect the RNA of DENV type 4 or Nounane virus. The alignment for the Flavi all S primer revealed mismatches at 6th and 8th position from the 3′-end which would have been rated as non-critical (Figure 2). However, only addition of DEN4 F primer improved the assay for DENV type 4 (Tables 1 and 2). Remarkably, the 3rd position from 3′-end mismatch in the reverse primer Flavi all AS2 for YFV, DENV type 2 and Usutu virus did not have any impact as these three viruses were detected well by Pan-Flavi assay (Figure 2). These results underline that the *in-silico* analysis provides only a limited prediction for the primer specificity, which still requires to be confirmed by laboratory testing. Designing of a single probe for detection of all flaviviruses is challenging because of the genetic diversity and the restricted length of conserved regions. One approach is the use of oligonucleotide mixtures covering many target sequence variants, which was applied for detection of DENV and Crimean-Congo Hemorrhagic fever virus (CCHF) [37,38]. Other approaches to design broad range probes are the use of a short conserved sequence in combination of minor groove binding (MGB) or LNA modifications [39,40]. First, we used 22 specific probes for flaviviruses generic detection, but the amplification signals were insufficient. To overcome this problem, probes with LNA bases were designed for the Pan-Flavi assay. Major advantages of LNA probes in comparison to other real-time PCR formats are the shorter probe length, stability, increased sensitivity, specificity and quenching efficiency which have been demonstrated by many studies [30,41-45]. One of three designed and tested LNA-based probes (Flavi all probe) successfully targeted a broad spectrum of different flaviviruses. The signal for detection of YFV and DENV type 4 were optimized by adding small fractions of specific probes (Table 5).

**Table 5:** Oligonucleotide sequence of primers and probes used in Pan-Flavi qRT-PCR assay

**Primer & Probe**	**Sequence**	**Concentration**	**Orientation**^**a**^	**Position**^**b**^	**Tm (°C)**
**Flavi all S**	TACAACATgATggggAARAgAgARAA	10 pmol	S	8993–9019	54.7
**DEN4 F**	TACAACATgATgggRAAACgTgAGAA	10 pmol	S	8996–9019	60.18
**Flavi all AS 1**	gTCCCANCCDgCKgTRTC	10 pmol	AS	9236–9253	52.5
	gTCCCATCCAgCKgTRTCATC	5 pmol	AS	9236–9256	57.1
**Flavi all AS 2**	gTgTCCCAgCCNgCKgTgTCATCWgC	10 pmol	AS	9232–9260	69.6
**Flavi all probe 1**	FAM-AARggHAgYMgNgCCA **+ T**H **+ T + g + g + T**-BBQ	5 pmol	S	9044–9065	69–83
**Flavi all probe 2**	FAM-Tg **+ g**TWYATgTggYTNg **+ g**RgC--BBQ	5 pmol	S	9062–9082	62–75
**Flavi all probe 3 mix**	FAM-Tg **+ g**TWYATgT **+ g**gYTNg **+ g**RgC—BBQ^c^	5 pmol	S	9062–9082	66-79
	FAM-CCgTgCCATATggTATATgTggCTgggAgC-BBQ^d^	0.5 pmol	S	9052–9081	74.7
	FAM-TTTCTggAATTTgAAgCCCTgggTTT-BBQ^e^	0.5 pmol	S	9086–9012	68
**Flavi all S2**	TACAACATgATgggMAAACgYgARAA	10 pmol	S	8996–9019	58.2
**Flavi all AS4**	gTgTCCCAGCCNgCKgTRTCRTC	10 pmol	S	9235–9260	64.1

Tm: temperature, Locked-Nucleic Acid bases are written as ‘ **+ _**’, e.g., **+A.**

Degenerate bases: R = (A/G), W = (A/T), K = (T/G), Y = (C/T), N = (A/G/T/C).

^*a*^ S: sense orientation, AS: antisense orientation.

^*b*^ YFV strain; accession no: NC 002031.

^*c*^ Flavi all probe, ^*d*^ Flavi probe YFV, ^*e*^ Flavi probe DEN4.

Final Pan-Flavi assay comprise of Flavi all S, Flavi all S2 & Flavi all AS4 primer and Flavi all probe 3 mix.

At present, RT-PCR in nested or hemi-nested format is used most frequently. It requires sequencing for the identification of viruses and needs approximately one day for experimentation. In addition, this carries a high risk of contamination caused by open handling of PCR products, increasing the potential for false positives. In contrast, the Pan-Flavi assay presented here requires only some 50 minutes for specific and sensitive detection of several flaviviruses in one reaction. The detection sensitivity of YFV, DENV, TBEV, JEV and WNV, using the described Pan-Flavi assay, was close to or as good as the species-specific qRT-PCR assays. These differences may have resulted from deviant reactions conditions, enzyme kits and instruments, which were used as published or in-house established, and it is to mention, that WNV and JEV assays were performed in a two-step PCR which is known to be more sensitive than the one-step procedure [46].

The sensitivity of the Pan-Flavi assay to detect RNA was demonstrated by using 10-fold serial dilutions of viral RNA from DENV-1, YFV and TBEV. The assay was able to detect 10–100 viral RNA copies per reaction, depending on the respective flavivirus. The assay allows the quantification of flavivirus RNA by use of standard plasmids or standardised in-vitro RNA, providing information regarding viral load and can be easily established in other laboratories. Therefore, the Pan-Flavi assay could be used to confirm the presence of flaviviruses in a sample without post amplification steps or complement species-specific flavivirus assays. The amplicon length is about 260 bp, which is sufficient to distinguish and identify different the virus by (pyro) sequencing or using for example an array.

The Pan-Flavi assay allows rapid detection of a broad range of flaviviruses including local-endemic and exotic species with a high level of sensitivity and is cost-effective, making it suitable for screening of livestock samples, monitoring viral activity, outbreak investigation as well as surveillance studies of flaviviruses, and might help finding and identifying novel flaviviruses. We expect that the assay can be also helpful for testing clinical samples from patients with symptoms especially in an early phase of infection and without knowledge about locally prevalent flaviviruses or travel history in order to avoid parallel testing on several flaviviruses, although a negative test will of course not prove absence of a flavivirus infection.

## Conclusions

Re-emergence of West Nile virus in Europe and continuous spread of certain flaviviruses such as dengue, yellow fever and Japanese encephalitis viruses represent a global danger to public health. The Pan-Flavi assay presented here has been developed and evaluated in comparison with flavivirus species-specific qPCR assays to detect a broad range of flaviviruses simultaneously in a single-tube. This assay represents a valuable tool for the detection of flaviviruses in livestock samples, and will support molecular diagnosis as well as surveillance studies of various vectors and non-human hosts.

## Methods

### Viruses and isolation of viral RNA

All flavivirus and non-flavivirus strains listed below were derived from cell culture and provided by the Robert Koch Institute, Berlin, Germany. The following inactivated and stable virus preparations were used in this study: DENV-1 VR344 (Thai 1958); DENV-2 VR345 (TH-36 strain); DENV-3 VR216 (H87 strain); DENV-4 VR217 (H241 strain); WNV Uganda strain (AY532665); WNV Israel (H. Bin, Sheba Medical Center, Israel), kunjin virus (Institute Pasteur, France), usutu virus (AY453411); JEV (ATCC SA14-14-2); Saint Louis encephalitis virus (SLEV) (ATCC VR-1265); TBEV strain K23 (AF091010); YFV strain 17D (X03700); YFV strain ASIBI (AY640589); YFV strain Brazil; YFV strain Ivory Coast; Russian Spring Summer Encephalitis virus (RSSEV); chikungunya virus (LR 2006); chikungunya virus African isolate; sindbis virus; Rift Valley Fever virus and influenza A virus subtype H5N1 (A/dk/Germany R603/06 H5N1).

Two different standard preparations of H1N1 influenza viruses (A/California/04/2009 and A/Hamburg/04/2009) used in this study were provided by the European Network for Diagnostics of Imported Viral Diseases (ENIVD). RNA of Nounane virus was kindly provided by Dr. Fabian Leendertz from NG2, Robert Koch Institute, Berlin. RNA of eleven African flaviviruses (Table 3) were kindly provided by Dr. Amadou A Sall and testing of these viruses was done at Institut Pastuer Dakar, Senegal.

Viral RNA from above described viruses was isolated from 140 μl aliquots of cell culture supernatants, using the QIAamp Viral Mini Kit (Qiagen, Hilden, Germany) according to the manufacturer’s instructions. RNA was eluted in 100 μl of elution buffer and stored at -80°C until further use.

### Primers and TaqMan probe

The flaviviral NS5 gene contains conserved regions often used for PCR testing. Our laboratory results but also the literature [27] revealed that the commonly used primers [26-28] exhibit a low sensitivity and we failed to detect rare flaviviruses such as Nounane virus. The primer binding region were aligned against all Genbank (NCBI) deposited flavivirus sequences including partial entries for the NS5 region only using the MEGA5 software. Based on the alignment, 32 primers and 22 probes representing all known variants of flavivirus sequences were selected to be used as mixture in one reaction (data not shown). In an alternative approach two degenerate generic primer sets and three degenerate probes containing LNA nucleotides were designed to match ‘all’ flaviviruses. LNA nucleotides contain a 2^′^–5^′^ bridge, increasing the duplex stability, which in turn allows the use of shorter sequence probes matching the conserved sequence motif.

### Pan-Flavi qRT-PCR assay

The Pan-Flavi qRT-PCR assay (Pan-Flavi assay) was run with the Transcriptor One-Step RT-PCR Kit (Roche Diagnostics Mannheim, Germany) according to the manufacturer’s instructions. In brief, 5 μl of RNA, 10 pmol of primers Flavi all sense, Flavi all antisense 2 and DEN4 F, and a mixture of three probes (Table 5) were added to the master mix. Optimized run conditions were as follows: reverse transcription at 55°C for 2 min, denaturation at 95°C for 30 s and 45 cycles of 95°C for 10 s and 60°C for 25 s. The amplification was performed on a Roche LightCycler 480 instrument, software version 1.5. The spectrum of the assay can be expanded by using Flavi all S2 instead of DEN4 F primer (Table 5).

Serial dilutions of a standard (10–10^6^ copies/μl) were run in duplicate for quantification. Standard plasmid was prepared by cloning the PCR product obtained from YFV using a TOPO TA Cloning Kit (Invitrogen, Karlsruhe, Germany) according to the manufacturer’s instructions and quantified by spectrophotometric method.

### Specific qRT-PCR for different flaviviruses

Except for DENV and JEV we used published group-specific assays for comparison.

For TBEV, we used an assay targeting the NS1 gene as described previously [32]. qRT- PCR was carried out in one-step format on an ABI 7500 cycler (Applied Biosystems, California, USA), using the Superscript III Platinum OneStep qRT-PCR Kit (Invitrogen).

A WNV-specific qRT-PCR assay for detection of lineages 1 and 2 was used to detect and quantify genome copies (GC) of WNV as described previously [31]. qRT-PCR was performed in two-step format on a Stratagene MX 3000 cycler (Agilent Technologies, Santa Clara, USA).

YFV-specific primer combination YFV FP/RP and probe YFV LNA2 were used to detect and quantify genomic RNA of YFV as described previously [30]. The assay was performed in one-step format on the LightCycler 480 instrument using the QuantiTect Virus Kit (Qiagen, Hilden, Germany).

DENV-genomic RNA was tested and quantified by an in-house qRT-PCR as described previously [29]. The assay is a DENV group-specific assay and was carried out in one-step format on an ABI 7500 real-time PCR system using the AgPath-ID One-Step RT-PCR Kit. Plasmid standards were used for the quantification of the DENV GE.

A JEV-specific in-house qPCR assay was used for detection and quantification of JEV GE. qPCR was performed in two-step format on a Stratagene MX 3000 cycler. cDNA was generated using SuperScript II (Invitrogen) and was stored at -20°C until use. The test was performed using Platinum Taq polymerase (Invitrogen) in a total volume of 25 μl containing 5 μl of cDNA, 2 μl of 10x Reaction Mix, 4 mM MgCl_2_, 300 nM of each primer (JEV9250 F: CgTCCAAAAgCTgggATACAT and JEV9334 R: gTCCCAYCCggCggTRTC), 100 nM of probe (FAM-TCCgTgAYATAgCAggRAAgCAAgg –BBQ) and 2.5 μM of dNTPs, using a Stratagene Mx3000 instrument and the following conditions: 15 min at 95°C; 45 cycles of 15 s at 95°C and 30 s at 60°C. Ten-fold serial dilutions of the standard (plasmid 10–10^6^ copies/reaction) were included in order to determine the limit of detection.

### Final protocol for extended range specificity

The pan-flavi assay was evaluated using forward primers Flavi all S and DEN4 F, reverse primer Flavi all AS2 and Flavi all probe 3 mix. Based on an alignment of latterly available NS5 gene flavivirus sequences, we substituted primer DEN4F by Flavi all S2 and Flavi all AS2 by Flavi all AS4 to include some novel flaviviruses (Table 3).

## Abbreviations

RT: Reverse transcription; Tm: Melting temperature; Ct: Threshold cycle; GC: Genome copies; DENV: Dengue virus; YFV: Yellow fever virus; TBEV: Tick-borne encephalitis virus; WNV: West nile virus; JEV: Japanese encephalitis virus; LNA: Locked-nucleic acids.

## Competing interests

The authors declare that they have no competing interests.

## Authors’ contributions

PP, MK conceived and designed the experiments; OL, MK, PP participated in the sequence alignment/assay design; PP, TK, UL, OF performed the Experiments; PP, OL, MK analysed the data; AS, OL, MN contributed reagents/materials/analysis tool, PP wrote paper. All authors have read and approved this manuscript.

## Authors’ information

Pranav Patel is working as a scientist at Department of Centre for Biological Security 1, Robert Koch Institute, Berlin, Germany and has vast experience on developing rapid diagnostic assays and platforms for detection of emerging pathogens.
